# Social attention: a possible early indicator of efficacy in autism clinical trials

**DOI:** 10.1186/1866-1955-4-11

**Published:** 2012-05-17

**Authors:** Geraldine Dawson, Raphael Bernier, Robert H Ring

**Affiliations:** 1 Autism Speaks, New York, NY, USA; 2 Department of Psychiatry, University of North Carolina, Chapel Hill, NC 27599-3366, USA; 3 Department of Psychology, University of Washington, Seattle, WA 98195, USA; 4 Department of Psychiatry, University of Washington, Seattle, WA 98195, USA; 5 Department of Pharmacology and Physiology, Drexel University School of Medicine, Philadelphia, PA 19102, USA

**Keywords:** Autism, Autism spectrum disorder, Social reward, Social attention, Oxytocin, Clinical trials, Behavioral intervention

## Abstract

For decades, researchers have sought to clarify the nature of the social communication impairments in autism, highlighting impaired or atypical 'social attention' as a key measurable construct that helps to define the core impairment of social communication. In this paper, we provide an overview of research on social attention impairments in autism and their relation to deficiencies in neural circuitry related to social reward. We offer a framework for considering social attention as a potential moderator or mediator of response to early behavioral intervention, and as an early indicator of efficacy of behavioral and/or pharmacological treatments aimed at addressing the social impairments in autism.

## Review

Recent conceptualizations of the diagnostic features of autism spectrum disorder (ASD) emphasize two core features: social communication and repetitive behaviors [1]. Decades of research have sought to clarify the nature of the social communication impairments, highlighting impaired or atypical 'social attention' as a key measurable construct that helps define the core impairment of social communication. In this paper, we provide a brief overview of behavioral and neuropsychological research on social attention impairments in autism and their relationship to deficiencies in neural circuitry related to social reward. We offer a framework for considering social attention as a potential moderator or mediator of response to early behavioral intervention, and a biomarker^a ^that could potentially be useful as an early indicator of efficacy of behavioral and pharmacological treatments aimed at addressing the social communication impairments in autism.

### Early manifestation of social attention impairments in ASD

Typical infants show preferential attention to people, including their eyes, faces, and movements from the first days of life. Faces, voices, and body movements are biologically relevant stimuli that are normally a strong focus of attention beginning early in life. Young children with autism, in contrast, pay less attention to other people and their actions and focus their attention instead on non-social objects [2]. Three to four-year-old children with ASD fail to show normal preferences for speech sounds [3-5]. Pierce *et al. *[6] reported that toddlers with ASD, aged 14 to 42 months, prefer to visually examine geometric images more than social images. Chawarska *et al. *[7] found that, unlike toddlers with typical development or developmental delay, toddlers with ASD did not have difficulty disengaging their attention from a face when presented with a competing stimulus. Impaired eye contact is an early emerging, cardinal feature of ASD present by at least 1 year of age in children with early onset autism [8,9]. A failure to orient to social stimuli, termed a 'social orienting impairment' was documented in preschool-age children with autism decades ago [10] and was subsequently demonstrated in 10-month-old infants who go on to develop autism [8]. More recently, Klin *et al. *[11] reported that 2-year-olds with autism orient to non-social contingencies rather than biological motion.

To help explain the impairment in social attention found in autism, Dawson and others have proposed the social motivation hypothesis, which posits that autism is associated with reduced social reward sensitivity manifest in a failure to affectively tag socially relevant stimuli [12-15]. Diagnostic criteria for autism describe 'a lack of spontaneous seeking to share enjoyment, interests, or achievements with other people' and 'lack of social or emotional reciprocity.' For example, preschool-age children with ASD are less likely to smile when looking at their mothers during social interaction [16], especially during joint attention episodes [17]. Related to this, Sung *et al. *[18] found evidence that a diminished social motivation trait (for example, seeking social activities and friendships) was heritable (heritability estimate = 0.19) in multiplex autism families.

It has further been hypothesized that reduced social attention has potentially negative downstream consequences for social and language development and learning, in general, affecting the development and specialization of neural circuitry subserving these domains, which is experience-dependent [19-21]. It has been hypothesized that early behavioral intervention can mitigate these negative consequences by enhancing social motivation by either stimulating nascent neural circuitry involved in social reward, or by co-opting neural reward systems that target non-social stimuli through classical conditioning (non-social reward, such as food or a toy, being paired consistently with a social stimuli, such as person in the context of treatment) [19].

### Neural mechanisms involved in reward processing and implications for ASD

Although there has been limited work conducted examining the neural correlates of reward processing in ASD, the existing findings from functional MR imaging, electrophysiological, and neuropsychological studies have informed our understanding of the neural mechanisms related to both social and non-social reward processing in ASD.

Functional imaging studies in typical populations have identified several key brain regions involved in reward processing. The anterior cingulate cortex (ACC) and orbitofrontal cortex (OFC), as well as regions involved in the mesolimbic dopamine system, which originates in the ventral tegmental area (VTA) and projects to the nucleus accumbens (NA) in the ventral striatum, have long been associated with reward processes. These regions have consistently been associated with the rewarding properties of alcohol and drugs [22], food [23,24], sex [25], and monetary gain [26-28]. Further, functional imaging studies implicate this system and these structures during the viewing of social stimuli such as faces [29-33] or when receiving social reinforcement [34].

Functional imaging studies with individuals with ASD have indicated differential functioning of these neural regions implicated in reward processing. Increased activation in the left ACC and left middle frontal gyrus was observed in a sample of high functioning adults with ASD during a target detection task yielding monetary rewards [35]. Further, the activation in the left ACC correlated with degree of social symptomology, as measured by parent interview, suggesting that disruptions in this structure of the reward system may contribute to the social deficits observed in ASD. Reductions in ACC volume and white matter have also been observed in ASD indicating structural differences in this region [36,37]. A recent study of 92 high-risk infant siblings demonstrated that those infants who eventually developed ASD exhibited diffuse aberrant development of white matter pathways between 6 and 24 months of age, based on diffusion tensor imaging. These results suggest that alterations in ACC white matter (as well as other white matter fiber tracts) may be present very early in life. School-aged children with ASD have demonstrated differential neural activity in response to monetary reward as well as social rewards. Children with ASD showed reduced neural activity in the ventral striatum in response to both monetary and social rewards provided during an abstract figure classification task. They demonstrated reduced activity in the ACC, the striatum, and ventral prefrontal cortex during the learning process but only in the social reward condition, not the monetary learning condition [38]. Children with ASD have also demonstrated reduced neural activity in these frontal circuits and the striatum compared to typically developing children during social cuing tasks, suggesting that social cues are not afforded the same neural importance in ASD as they are for typical children [29]. However, typical activation of the nucleus accumbens has also been observed in adults with ASD when the reward stimulus is an object of specific interest. Dichter and colleagues [39] rewarded participants with either money or pictures of favored objects (such as trucks and mechanical devices) in response to quick button-press responses to a bulls-eye target. When anticipating or receiving a monetary reward, ASD adults showed reduced activation in the nucleus accumbens compared to controls. However, when the object reward was provided, the ASD adults showed a similar level of reward system activation to the typical controls. The limited imaging findings in the literature indicate differential activation in reward-related neural structures in ASD for a variety of stimulus types and notably for social stimuli.

Electrophysiological studies have also revealed atypical functioning of the reward system in ASD in relation to processing social or non-social stimuli. EEG provides insight into the temporal dynamics of brain activity that functional magnetic resonance imaging (MRI) studies are unable to elucidate. Event-related potentials (ERPs) can be derived from EEG recordings, and reflect the averaged brain response to a single stimulus event that is repeatedly presented. Several measures can be derived from the ERP, including the latency and amplitude of the different positive- and negative-going peaks of the ERP wave form. Different peaks of the wave form reflect different processes, such as attention, memory, expectation, and so on.

An incentivized go/no-go task adapted for ERP studies has provided a paradigm for examining reward anticipation as both social and monetary rewards increase the accuracy of the inhibited response in typical individuals [40]. The amplitude of the P3, a positive-going peak occurring approximately 300 ms after stimulus onset, has been used as an indicator of motivational salience, with greater amplitudes indicative of increased reward value [41,42]. On an incentivized go/no-go task, children with ASD showed an attenuated P3 amplitude response to both social (as indicated by a picture of a smiling face) and non-social (monetary) rewards, suggesting a reward-processing deficit, but not one specific to social stimuli [42]. Feedback-related negativity (FRN), an ERP response marked by greater negative amplitude in response to a loss, such as loss of money, than a gain, has been shown to be typical in ASD. Individuals with ASD show expected greater negative amplitude to monetary losses compared to gains during learning tasks or guessing games [43,44]. This FRN result contrasts with findings in individuals with ASD of attenuated amplitudes of event-related negativity (ERN) which is a negative going wave that occurs within 100 ms of making an incorrect response on a task [45,46], although greater amplitudes have been noted in ASD as well [47]. Larson and colleagues suggest that this discrepancy of findings between FRN and ERN suggest that it is not the valence of the feedback, but the source of the feedback, such as social or non-social, that is the critical component [44].

Neuropsychological studies in individuals with ASD have also yielded insight into the mechanisms for social and non-social reward processing. On neuropsychological tasks reflecting functioning of ventromedial prefrontal cortex (VMPFC) and dorsolateral prefrontal cortex (DLPFC), children with ASD showed similar levels of performance relative to mental-age matched typical children and children with developmental delay [48]. The VMPFC tasks included delayed non-matching to sample (DNMS) and object discrimination reversal (ODR), both tasks shown to tap the VMPC in non-human primate studies. The DLPFC tasks included delayed response tasks and spatial reversal. Only performance on the VMPFC task was correlated with severity of core autism symptoms (joint attention ability). Given the relationship between the VMPFC and reward processing regions such as the OFC, this neuropsychological finding provided early evidence for a disruption in reward processing in ASD. Additionally, performance on neuropsychological tasks that measure learning of reward associations, such as DNMS and ODR, predict social and communication growth rates in children with ASD [49]. High functioning 6 to 7-year-olds with ASD performed more poorly on a 'hot' executive function (delayed gratification) task but not a 'cold' executive function (dimensional change card sort) task than age-matched typical peers, indicating that executive functioning tasks that rely more heavily on the reward pathway are more challenging for children with ASD [50]. These behaviorally-based testing results provide further insight into the neural mechanisms for reward processing and offer additional evidence of differential processing of social and non-social rewards in ASD.

### Neuropeptides involved in reward processing and ASD

Advances in research on prosocial neuropeptide systems of the central nervous system (CNS) have offered additional insights into the molecular and cellular mechanisms involved in reward processes supporting social behaviors, and may offer specific clues to the importance of these systems to the development of social impairments in ASD. In this area of research, particular attention has focused on evidence from studies of the evolutionarily related nonapeptides oxytocin (OT) and vasopressin (AVP) [51]. Across mammalian species from rodents to humans, OT and AVP have been shown to be powerful modulators of neural activity that regulate a diverse range of CNS functions in both males and females in a manner physiologically distinct from the well-described endocrine activities of these molecules [52,53]. In the context of reward processing, neuroanatomical, biochemical, and behavioral evidence have emphasized the relevance of functional interactions between oxytocinergic and dopaminergic neurotransmitter systems of the CNS in social cognition and behavior [54]. More specifically, a network of oxytocinergic-dopaminergic neural circuitry suggests a mechanism by which OT recruits reward and reinforcement to enhance the salience of social stimuli [55]. Individual variation in maternal behaviors toward infants, and the involvement of brain reward circuitry, appear to be intrinsically linked with the development of central oxytocinergic and dopaminergic systems [56]. Utilizing BOLD MRI in rodents, OT administration mimics activation of the same brain areas involved in olfactory, emotional, and reward processing that are observed postpartum in dams during suckling, which can be antagonized pharmacologically by administration of an OT receptor (OXTR) antagonist [57]. Even in nulliparous females, OT administration increases the functional connectivity between key CNS structures involved in reward processing following exposure to recordings of infant laughter, providing additional support that OT acts to enhance the salience of social stimuli [58]. Combinatorial methods involving genomic approaches and multimodal neuroimaging of human adults revealed a relationship between genetic variation in the gene encoding the OT receptor (*OXTR*), and differences in reward dependence as measured with the Tridimensional Personality Questionnaire [59]. Collectively, the extant evidence to date would suggest that prosocial neuropeptides such as OT engage reward circuitry of the CNS to support effects on social functioning, and implicates this functional connectivity in the etiology of underlying social deficits in ASD.

#### Studies showing altered levels of oxytocin in ASD

A hypothesis of oxytocinergic deficiency in ASD has emerged, and is supported by different evidence from fields of biochemical and genetic research. Reduced circulating levels of OT in plasma have been reported in children with autism when compared to typically developing children, a finding that is correlated with greater impairment in social skills [60,61]. Lower levels of OT were associated with lower scores on social and developmental measures of behavior. Abnormalities in the proteolytic processing of the inactive precursor peptide of OT, which is required for the production of biologically active peptide, have also been observed in individuals with autism and associated with lower circulating levels of OT [61]. This suggests a diversity of risk factors may conspire to adversely impact oxytocinergic function in ASD. From a different perspective, numerous genetic studies have revealed that variation in *OXTR *may be also specifically associated with ASD. Adding to this, combined analysis of linkage data from two independent genome-wide screens of the Autism Genetic Resource Exchange (AGRE) and a large Finnish autism cohort identified *OXTR *among four susceptibility loci for autism [62]. Evidence of association between *OXTR *genotype and ASD have been observed in most, but not all studies [63,64]. Of interest, evidence of allelic association between *OXTR *and ASD has been observed across ethnic backgrounds including Caucasian [65], Chinese [66], and Japanese populations [67]. With respect to social functioning in ASD, a clear association between *OXTR *genotype and social endophenotypes has been established in a large family-based study involving 2,333 individuals [68]. These data are consistent with evidence from smaller studies suggesting that variation in *OXTR *plays an important role in influencing the development of communication, daily living skills, and socialization in individuals with autism [69]. Intriguingly, variation in genes encoding proteins biologically coupled to oxytocinergic function has also been associated with ASD. For example, allelic variants in the *CD38 *gene, encoding a protein involved in the secretion of OT from hypothalamic neurons, have been identified in individuals with ASD and are associated with reduced plasma levels of OT [70]. Examination of postmortem brain tissue from individuals with ASD has revealed expression differences in *OXTR *that appear tied biologically to altered expression of specificity protein 1 (*SP1*), a transcription factor involved in the expression of several ASD candidate genes including *OXTR *[71]. Changes in the methylation status of the *OXTR *promoter have also been associated with altered expression of the receptor in the postmortem brains of persons with autism, suggesting that epigenetic mechanisms may also be complicit in pathogenic regulation of *OXTR *expression in ASD [72]. Collectively, a growing body of evidence is accumulating that reduced oxytocinergic function may represent an important contributing factor to an endophenotype underlying social deficits in ASD.

#### Impact of oxytocin on social attention/functioning in ASD participants

Numerous clinical studies have directly investigated the impact of OT on social functioning in humans, including trials with OT in individuals living with ASD, and have provided the most compelling evidence to date supporting a proof of concept for oxytocinergic system involvement in social functioning. These studies have largely involved the experimental use of intranasally administered OT, a synthetic preparation of the peptide previously developed and approved for use with non-CNS indications (for example lactation support) [73]. In healthy human volunteers, a broad range of effects have been described for OT administration on social cognition, including improvements in the encoding and recognition of facial expression [74], increased empathic perception [75,76], enhanced memory encoding of faces in humans, but not of non-social stimuli [77] and responses to biological motion [76]. Additionally, OT enhances socially-reinforced learning [75], promotes trust [78], enhances the subjective perception of attachment [79], and increases cooperative behavior with social cues [80]. Challenge studies in healthy volunteers also reveal pharmacodynamic effects of OT on neural activity in many of the same CNS structures where aberrant activity has been observed in ASD versus neurotypical controls. For example, OT increases functional connectivity between the amygdala and the ACC, which suggests that this peptide acts simultaneously to enhance neural control over negative emotionality and increase the incentive salience of social stimuli such as infant laughter [58]. It is possible that OT may act at the level of specific circuits, in a compensatory manner, to address deficits in neural activity observed in ASD.

Implicit in the observed effects of OT on social functioning of typically developing individuals is the translational potential for oxytocin-based therapeutics as a treatment option for addressing core social deficits in ASD [81], and several small clinical trials have directly investigated the clinical efficacy of OT in individuals with ASD. Investigating comprehension of affective speech in adults with autism or Asperger syndrome, Hollander *et al. *demonstrated that infusion of OT could significantly enhance the processing and retention of social information [82]. Other studies found that OT increased social engagement in ASD participants. Using a social interaction task, where participants with autism engage in a simulated ball-toss game over a computer network with three fictitious partners, Andari *et al. *demonstrated that intranasal OT increased social approach and social comprehension [83]. Intranasal OT administration also improves emotional recognition in children with ASD participating in a Reading the Mind in the Eyes Test-Revised, one of the most widely used tasks for examining the Theory of Mind [84].

In summary, there is evidence that ASD is associated with oxytocinergic deficiency which may underlie deficits in social motivation and engagement. Specifically, ASD is hypothesized to involve deficiencies in the network of oxytocinergic-dopaminergic neural circuitry by which OT recruits reward and reinforcement to enhance the salience of social stimuli. Early trials involving administration of OT have shown promising results for enhancing social approach and comprehension in ASD participants.

### Social attention as a moderator and mediator in autism clinical trials

In light of neurophysiological, behavioral, and molecular evidence that autism is associated with reduced activity of social reward circuitry which are hypothesized to underlie deficits in social motivation in ASD, there is great interest in developing feasible, valid biomarkers reflecting degree of social motivation that could be used as early indicators of efficacy in clinical trials aimed at addressing the social impairments in autism. Many studies have demonstrated that reward facilitates attention to specific stimuli, and that reward-based priorities strongly influence how attention is allocated [85-94]. For example, ERP measures of attention (for example P3 ERP amplitude) have been shown to be closely linked to reward anticipation [42], and other studies have demonstrated that visual attention to a stimulus is modulated by its associated value [95,96]. Research has also shown that the magnitude of visual attention that is created by reward is predicted by the response to reward feedback in the ACC [89].

Measures of social attention have shown promise as early predictive diagnostic biomarkers for ASD [6]. Here, we argue that such measures could also serve as both an early sign of efficacy and for stratification in clinical trials designed to enhance social communicative behavior. A distinct advantage of measures of social attention is their feasibility with participants of a wide age range (infants to adults) and ability levels (non-verbal and intellectually disabled to normal cognitive functioning). We first consider how such measures might be considered in the context of a clinical trial testing the efficacy of an early behavioral intervention.

**Figure 1:**
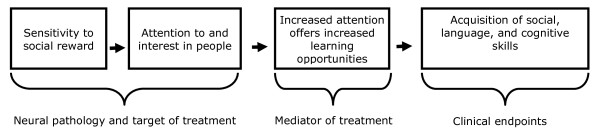
**Role of social attention as an early indicator of efficacy in clinical trials**.

Vismara and Rogers [97] recently summarized the extensive research literature on behavioral interventions for children with ASD and concluded that both comprehensive and targeted behavioral interventions can be effective in improving communication, social skills, and management of problems behavior for young children with ASD. Recent studies suggest that relatively brief targeted interventions can significantly improve autism symptoms in young children and toddlers with ASD [98-100]. As described by Dawson [19] and as illustrated in Figure 1, early behavioral intervention serves to alter children's sensitivity to social reward and, thereby, altering levels of social attention. Increases in social attention greatly enhance opportunities for learning, serving as a mediator of the effects of early intervention on later outcomes that can be measured by standardized tests of cognitive, language, and adaptive behavior. As such, measures of social attention could potentially serve as an early predictor of treatment response in intervention trials, whether behavioral or pharmacological, in which enhancement of social motivation/social attention is presumed to be affected and central to the mechanism of change.

**Figure 2:**
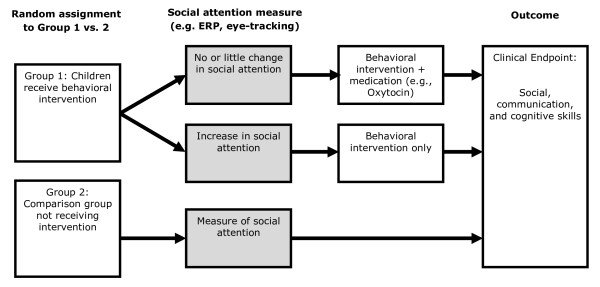
**Social attention as an early indicator of efficacy in a clinical trial testing combined behavioral and pharmacological treatment**.

One of the challenges of clinical trials in autism is the great variability in responses to intervention. With respect to behavioral interventions, it is well-established that there is great individual variability in outcomes, with some children showing dramatic and rapid gains and others progressing more slowly. For the latter group, it is possible that response to a behavioral intervention could be enhanced through pharmacological intervention that augments social attention or otherwise improves the tractability of other components of the treatment plan. A hypothetical adaptive study design for such an intervention trial is shown in Figure 2. In this instance, it is suggested that measures of social attention could potentially serve as a biomarker for stratification into two arms of a clinical trial (behavioral intervention augmented with a pharmacological intervention *vs*. behavioral intervention alone). If sample size would permit, the design could involve randomization of the subgroup showing no increase in social attention into either (1) continuation with behavioral intervention alone versus (2) behavioral intervention plus pharmacological treatment.

### How to measure social attention in individuals with ASD

Given the primacy of social attention in the deficits observed in ASD and its relationship to subsequent development, the careful assessment and characterization of social attention impairments in ASD may provide insight into which children will respond positively to interventions that are dependent upon some degree of social attention. Social attention is strongly related to joint attention skills, and through joint attention, to subsequent language development [101]. Social attention remains relatively stable in early years [102], has potential diagnostic predictive power [6], and can be assessed at the behavioral and electrophysiological levels. A variety of behavioral, electrophysiological, and eye tracking measures have been used to assess social attention abilities in individuals with ASD. Several have promise as easy to administer, objective, and informative measures for potential use as predictive indicators of response to treatment. These measures are summarized in Table 1 and described below.

**Table 1:** Selected methods for measuring social attention in individuals with ASD

	Task description	Sample	Significant findings	Admin time
*Behavioral measures*				

Social Orienting Task (Dawson *et al*., 1998)	Child is presented social (for example calling name, clapping hands) and non-social (such as car horn, kitchen timer) sounds from four locations around the room. Stimuli are presented for 6 s at matched decibel levels, and delivered once in the child's left and right visual field and once 30° behind the child to the left or right. The frequency with which the child orients to the sound is tallied	20: 4 to 6-year-olds with ASD 19: 4 to 6-year-olds with DS 20: verbal mental age-matched TYP controls	ASD group oriented less to all stimuli than controls. This was more pronounced for social stimuli	15 to 20 min

Social Orienting Continuum and Response Scale (Mosconi *et al*., 2009)	SOC-RS provides ratings for four behaviors related to social orienting: social referencing, joint attention, orienting to name, and social smiling, coded based on videotaped recording of standardized activities administered during the Autism Diagnostic Observation Schedule (ADOS). Behaviors are coded as rate/min and converted to z scores, except orienting which is scored as the trial number which orienting occurs. A total score is compiled by averaging standardized ratings	53: 18 to 35-month-olds with ASD; 27 of whom participate 24 months later for follow-up 35: age-matched TYP controls (15 at Time 1 and 20 at Time 2)	Deficits in social referencing, joint attention, and orienting to name observed at 2 years persisted at 4 years of age	30 to 45 min (length of ADOS)

Visual Preference Task (Pierce *et al*., 2011)	Child observes a 1-min video with one side showing geometric patterns and the other side showing children in movement (for example yoga). Total fixation time within each movie type is tallied	37: ASD toddlers 22: DD toddlers 51: TYP toddlers	Toddlers with ASD spent more time fixating on geometric patterns	1 min

Auditory Preference Task (Kuhl *et al*., 2005)	Child is presented with child-directed speech sounds or frequency and amplitude matched non-speech sounds from speakers placed to the left and right of the child. The number of head turns to the side presenting non-speech sounds is tallied	29: 2.5 to 4.5-year-olds with ASD 29: 1 to 4-year-olds mental age-matched TYP controls	ASD group showed greater preference for non-speech sounds than controls	5 min

*Electrophysiological measures*				

Habituation Task (Guiraud *et al*., 2011)	Child is presented with two different infrequent (11.5% occurrence each) sounds (650 Hz pure tone and white noise) randomly within a sequence of 500-Hz pure tones. Approximately 500 trials are presented. Sound intensity is 70 dB SPL and duration is 100 ms with an inter-stimulus interval of 700 ms. Average amplitude of P150 recorded from central electrodes for repeated and infrequent tones is calculated as measure of neural habituation	35: 9-month-olds with ASD siblings (high risk) 21: 9-month-olds with TYP siblings (low risk)	High-risk infants showed less habituation to repeated tones and reduced sensitivity to changes in tones	10 min

Face N170 Task (Dawson *et al*., 2004; Webb *et al*., 2006)	Child is presented with static pictures of faces (neutral or fear expressions; familiar and unfamiliar) of 500 ms duration followed by a 500-1000 ms inter-trial interval. Fifty trials of each condition are presented. Average amplitude and latency of prN170, N300 recorded and amplitude of Negative Slow Wave (NSW) from posterior electrodes for all conditions is calculated	29: 3 to 4-year-olds with ASD 22: age-matched TYP controls	ASD children had longer latency to prN170 and failed to show differential amplitude of the N300 and NSW between conditions	10 min

*Eye tracking measures*				

Visual Attention Task(Klin *et al*., 2002)	Individual views videotape clips of complex social situations while visual fixation patterns are recorded	15: teens and adults with ASD 15: age and verbal IQ matched TYP controls	Individuals with ASD attended less to faces and more to objects relative to controls	10 min

Spontaneous eye blinking (Shultz *et al*., 2011)	Toddlers view a video showing physical movements of objects (for example door on toy wagon) and affective social interactions (such as an argument between children). Instantaneous blink rate and timing of blink inhibition as a function of viewer engagement and stimulus type is recorded	41: 2-year-olds with ASD 52: age-matched TYP controls	TYP toddlers inhibited blinking earlier than ASD toddlers indicating reduced anticipation of upcoming salient information in ASD	2 min

ASD, autism spectrum disorder; DS, Down syndrome; DD, developmental delay; TYP, typical development; FRAX, fragile X syndrome.

#### Behavioral measures

At the behavioral level a number of assessment tasks are appropriate for use with young children with ASD. In the social orienting task [10] a child is presented with a variety of auditory stimuli while engaged with an experimenter at a table. During the task the child and experimenter sit across from each other at a table while a second experimenter delivers a variety of social (such as calling the child's name, clapping hands) and non-social (such as car horn honking, kitchen timer) sounds from four locations around the room. Each stimulus lasts approximately 6 s, is matched on decibel level, and is delivered once in the child's left and right visual field and once 30° behind the child to the left or right. The frequency with which the child orients to the sound is tallied. Children are also prompted by the examiner to jointly attend to an object (a star) posted in the four identified locations throughout the testing room. The prompt is both verbal ('look') and visual (point) and the number of correctly followed joint attention bids is tallied. Children with ASD, compared to typical peers and children with Down syndrome, more frequently failed to orient to all stimuli on the social orienting task with greater impairment for the social stimuli and showed greater joint attention impairments. Those children with ASD that did orient to the social stimuli showed delays in doing so relative to the comparison groups [10]. Additionally, impairments on the social orienting task, along with impairments in joint attention, best distinguished children with ASD from same age typical peers and peers with developmental delay [101].

The Social Orienting Continuum and Response Scale (SOC-RS) is a behaviorally based coding measure that allows for the quantification of social orienting abilities that are observed during the administration of a structured play session, the Autism Diagnostic Observation Scale (ADOS) [102]. During the administration of the ADOS several presses and activities are utilized in a standardized way to assess a child's response to his or her name, response to joint attention bids, and the frequency and quality with which a child initiates joint attention. The SOC-RS provides ratings for four behaviors related to social orienting, including social referencing, joint attention, orienting to name, and social smiling that are coded based on the observation of a videotaped recording of an ADOS administration. In a longitudinal sample of 2 to 4-year-olds with ASD, Mosconi and colleagues found impairments in social referencing, joint attention, and orienting to name relative to typical peers at 2 years of age and the same impairments along with deficits in social smiling, the fourth domain, when the children were 4 years of age [102]. There was no change over time in the social orienting composite score derived from the four domains assessed indicating robust impairments in social orienting over time for children with ASD.

Visual and auditory preference tasks are other behavioral measures that assess social orienting abilities in ASD and that could prove informative as a predictive indicator of subsequent treatment response. In preferential looking tasks, two visual images or types of images, such as social scenes or toys, are simultaneously presented to a child and the total time the child spends looking at each image is tallied. A percentage of looking time to each type of image can then be calculated as an indicator of visual preferences. When presented with 1 min videos of moving geometric patterns displayed on one side of a monitor and children doing yoga on the other, toddlers with ASD ranging from 14 to 42 months spent more time looking at the geometric patterns relative to the social scenes than same age typical peers and peers with developmental delay [6]. Further, the positive predictive value for classifying a toddler with ASD was 100% if the toddler spent more than 69% of the time watching the geometric pattern. Auditory preference tasks involve the presentation of sounds, such as speech and non-speech sounds, via speakers placed on alternate sides of a child. The number of head turns in the direction of the two stimuli types can be tallied. Kuhl and colleagues utilized an auditory preference task in which young children with autism and typical peers oriented to a loudspeaker to the left and right that presented either child-directed speech sounds or frequency and amplitude matched non-speech sounds [5]. During four familiarization trials in which the sound types were alternated, a light atop one of the loudspeakers was turned on and when the child oriented to the light, the sound was presented. The side on which the sound type was presented was counterbalanced. During testing trials, when the child made a 30° head turn toward the light, the sound was activated. The number of head turns to the side presenting non-speech sounds was tallied. Young children with ASD showed a greater preference for the non-speech sounds than their typical peers. Further, when the children with ASD were divided into two groups (a group that preferred non-speech stimuli and a group that preferred speech stimuli), those preschoolers that did orient to speech sounds demonstrated more typical electrophysiological functioning as measured with an ERP index of stimulus change processing.

#### Electrophysiological measures

The use of electrophysiological measures, such as the ERP response to faces, is another potential early indicator of efficacy. Pre-pulse inhibition, for example, has been proposed as an outcome measure in clinical trials in individuals with fragile X syndrome [103]. ERP paradigms do not rely on language or behavioral responses beyond passive viewing, making these paradigms excellent for infants or children of all functioning levels.

Habituation tasks might reflect social attention processes. In habituation paradigms, the repeated presentation of a stimulus results in decreased attention to that stimulus, providing insight into the perceptual and cognitive abilities of young children. High-risk infants (infant siblings of children with ASD) showed decreased habituation to repeated presentations of pure tones as indexed by an early ERP component relative to same-age peers with typically developing older siblings. Further, when presented with a deviant auditory stimulus, the high-risk infants did not show the same amplitude increase of the ERP component as their low-risk peers [104]. This reduced habituation to repeated stimuli and the corresponding attenuated response to stimulus change may play a role in the reduced sensitivity to social stimuli and the orienting deficits observed in ASD.

Face-related ERPs also could reflect social attention processes [12]. Such paradigms involve the presentation of faces, either upright or inverted, with neutral or emotional expressions, or that are familiar or unfamiliar, along with the presentation of comparison stimuli, such as toys, cars, or houses. Latency and amplitude of select ERP components, such as the face specific N170, can then be analyzed. Compared to typically developing and developmentally delayed peers, individuals with ASD fail to show amplitude changes in negative going waves approximately 300 ms after the presentation of neutral and fearful faces [105,106] and show increased latencies in the early negative going N170 component in response to the observation of upright and inverted faces [107]. Indeed, a computerized face-training intervention has been shown to modulate the ERP response to faces in adults with ASD, underscoring the utility of ERP paradigms as a measure of treatment response [108]. Adults with ASD underwent an 8-week face expertise training intervention with ERP and behavioral assessments conducted before and after intervention. The intervention resulted in behavioral improvements in face recognition and modulated the P1 amplitude in response to viewing faces [108].

#### Eye tracking measures

Eye-tracking technology provides another avenue to assess social attention in ASD. Eye-tracking is being actively explored as an outcome measure in ASD clinical trials (for example, http://www.clinicaltrials.gov/ct2/show/NCT01425918?term=eye-tracking&rank=7). Through cameras that non-invasively capture the movement of the eye, measurements of viewing patterns can be recorded and time spent fixating or looking at parts of static images or places in a dynamic scene can be quantified for analysis. Pioneering work assessing gaze and fixation patterns in individuals with ASD indicated decreased attention to faces and increased attention to objects in social scenes with the added finding that the amount of time spent looking at objects correlated with social impairment [109]. Further work combining eye-tracking technology with a preferential looking paradigm has indicated that toddlers with ASD fail to show a preference for point-light depictions of biological motion over scrambled point light motion as typical toddlers do [11].

Shultz, Klin, and Jones [110] recently reported on a novel measure of social attention/engagement derived from eye-tracking paradigms. They measured spontaneous eye blinking in toddlers with ASD and those with typical development while the toddlers watched a videotape containing segments displaying primary physical motion versus emotionally-laden interactions between two other toddlers. They found that both groups modulated the timing of blink inhibition when watching the tape, compared to a baseline period. Whereas typical toddlers showed greater blink inhibition during the social scene than during the non-social scene, toddlers with ASD showed the reversed pattern. Measures of blink inhibition can potentially serve as indices of perceived stimulus salience and can, therefore, be helpful measures of social attention/engagement in young children with ASD. Future studies are needed to determine whether the patterns of blink inhibition found by Shultz *et al. *are consistent throughout development.

## Conclusions

Autism is characterized by early-emerging impairments in social attention believed to be related to a reduced sensitivity to the reward value of social stimuli. Such impairments in social attention can have substantial detrimental impact on subsequent learning and neural development and specialization. Early behavioral intervention serves to increase children's attention to and enjoyment of social interactions, thereby increasing opportunities for learning and helping steer brain and behavioral development back toward the normal trajectory [19]. Oxytocin may enhance social engagement and attention in persons with ASD through its effects of neural circuitry related to social reward. Attention is closely related to the reward value of stimuli, activating the ACC which is known to mediate attention and be a key region involved in reward processing. We have hypothesized that measures of social attention could serve as a moderator or mediator in autism clinical trials, and may serve as an early read-out of efficacy and as a means of decision-making in an adaptive trial. Future research will be needed to validate the utility of social attention when used in this manner.

## Endnotes

^a^A biomarker has been defined as any characteristic that is objectively measured and evaluated as an indicator of normal biological processes, pathogenetic processes, or pharmacological responses to a therapeutic intervention. Thus, biomarkers can be behaviors or physiological traits that indicate early response to an intervention and need not be a biological measure. Social attention can be measured using behavioral (such as eye-tracking) or physiological (such as event-related potentials) indices.

## Abbreviations

ACC: Anterior cingulate cortex; ADOS: Autism Diagnostic Observation Scale; ASD: Autism spectrum disorder; AVP: Vasopressin; CNS: Central nervous system; DLPFC: Dorsolateral prefrontal cortex; ERN: Event-related negativity; ERP: Event-related potential; FRN: Feedback-related negativity; NA: Nucleus accumbens; MRI: Magnetic resonance imaging; OFC: Orbital frontal cortex; OT: Oxytocin; OXTR: Oxytocin receptor; SOC-RS: Social Orienting Continuum and Response Scale; VMPFC: Ventromedial prefrontal cortex; VTA: Ventral tegmental area

## Competing interests

The authors declare that they have no competing interests.

## Authors' contributions

GD, RB, and RR contributed equally to the writing of this paper. All authors read and approved the final manuscript.

## References

[B1] Lord C Petkova E Hus V Gan W Lu F Martin DM Ousley O Guy L Bernier R Gerdts J Algermissen M Whitaker A Sutcliffe JS Warren Z Klin A Saulnier C Hanson E Hundley R Piggot J Fombonne E Steiman M Miles J Kanne SM Goin-Kochel RP Peters SU Cook EH Guter S Tjernagel J Green-Snyder LA Bishop S A multisite study of the clinical diagnosis of different autism spectrum disorders Arch Gen Psychiatry 2012 69 306 313 10.1001/archgenpsychiatry.2011.148 22065253 PMC3626112

[B2] Shic F Bradshaw J Klin A Scassellati B Chawarska K Limited activity monitoring in toddlers with autism spectrum disorder Brain Res 2011 1380 246 254 21129365 10.1016/j.brainres.2010.11.074 PMC3050079

[B3] Klin A Young autistic children's listening preferences in regard to speech: a possible characterization of the symptom of social withdrawal J Autism Dev Disord 1991 21 29 42 10.1007/BF02206995 1828067

[B4] Klin A Listening preferences in regard to speech in four children with developmental disabilities J Child Psychol Psychiatry 1992 33 763 769 10.1111/j.1469-7610.1992.tb00911.x 1376327

[B5] Kuhl PK Coffey-Corina S Padden D Dawson G Links between social and linguistic processing of speech in preschool children with autism: behavioral and electrophysiological measures Dev Sci 2005 8 F1 F12 10.1111/j.1467-7687.2004.00384.x 15647058

[B6] Pierce K Conant D Hazin R Stoner R Desmond J Preference for geometric patterns early in life as a risk factor for autism Arch Gen Psychiatry 2011 68 101 109 10.1001/archgenpsychiatry.2010.113 20819977 PMC4894313

[B7] Chawarska K Volkmar F Klin A Limited attentional bias for faces in toddlers with autism spectrum disorders Arch Gen Psychiatry 2010 67 178 185 10.1001/archgenpsychiatry.2009.194 20124117 PMC4871149

[B8] Werner E Dawson G Osterling J Dinno N Brief report: Recognition of autism spectrum disorder before one year of age: a retrospective study based on home videotapes J Autism Dev Disord 2000 30 157 162 10.1023/A:1005463707029 10832780

[B9] Zwaigenbaum L Bryson S Rogers T Roberts W Brian J Szatmari P Behavioral manifestations of autism in the first year of life Int J Dev Neurosci 2005 23 143 152 10.1016/j.ijdevneu.2004.05.001 15749241

[B10] Dawson G Meltzoff AN Osterling J Rinaldi J Brown E Children with autism fail to orient to naturally occurring social stimuli J Autism Dev Disord 1998 28 479 485 10.1023/A:1026043926488 9932234

[B11] Klin A Lin DJ Gorrindo P Ramsay G Jones W Two-year-olds with autism orient to non-social contingencies rather than biological motion Nature 2009 459 257 261 10.1038/nature07868 19329996 PMC2758571

[B12] Dawson G Webb SJ McPartland J Understanding the nature of face processing impairment in autism: insights from behavioral and electrophysiological studies Dev Neuropsychol 2005 27 403 424 10.1207/s15326942dn2703_6 15843104

[B13] Dawson G Carver L Meltzoff AN Panagiotides H McPartland J Webb SJ Neural correlates of face and object recognition in young children with autism spectrum disorder, developmental delay, and typical development Child Dev 2002 73 700 717 10.1111/1467-8624.00433 12038546 PMC3651041

[B14] Grelotti DJ Gauthier I Schultz RT Social interest and the development of cortical face specialization: what autism teaches us about face processing Dev Psychobiol 2002 40 213 225 10.1002/dev.10028 11891634

[B15] Waterhouse L Fein D Modahl C Neurofunctional mechanisms in autism Psychol Rev 1996 103 457 489 8759044 10.1037/0033-295x.103.3.457

[B16] Dawson G Hill D Spencer A Galpert L Watson L Affective exchanges between young autistic children and their mothers J Abnorm Child Psychol 1990 18 335 345 10.1007/BF00916569 2376657

[B17] Kasari C Sigman M Mundy P Yirmiya N Affective sharing in the context of joint attention interactions of normal, autistic, and mentally retarded children J Autism Dev Disord 1990 20 87 100 10.1007/BF02206859 2139025

[B18] Sung YJ Dawson G Munson J Estes A Schellenberg GD Wijsman EM Genetic investigation of quantitative traits related to autism: use of multivariate polygenic models with ascertainment adjustment Am J Hum Genet 2005 76 68 81 10.1086/426951 15547804 PMC1196434

[B19] Dawson G Early behavioral intervention, brain plasticity, and the prevention of autism spectrum disorder Dev Psychopathol 2008 20 775 803 18606031 10.1017/S0954579408000370

[B20] Johnson MH Griffin R Csibra G Halit H Farroni T de Haan M Tucker LA Baron-Cohen S Richards J The emergence of the social brain network: evidence from typical and atypical development Dev Psychopathol 2005 17 599 619 16262984 10.1017/S0954579405050297 PMC1464100

[B21] Marcus DJ Nelson CA Neural bases and development of face recognition in autism CNS Spectr 2001 6 36 59 17008831 10.1017/s1092852900022872

[B22] Robinson TE Berridge KC The neural basis of drug craving: an incentive-sensitization theory of addiction Brain Res Brain Res Rev 1993 18 247 291 8401595 10.1016/0165-0173(93)90013-p

[B23] Ettenberg A Camp CH Haloperidol induces a partial reinforcement extinction effect in rats: implications for a dopamine involvement in food reward Pharmacol Biochem Behav 1986 25 813 821 10.1016/0091-3057(86)90392-8 3786340

[B24] O'Doherty JP Deichmann R Critchley HD Dolan RJ Neural responses during anticipation of a primary taste reward Neuron 2002 33 815 826 10.1016/S0896-6273(02)00603-7 11879657

[B25] Melis MR Argiolas A Dopamine and sexual behavior Neurosci Biobehav Rev 1995 19 19 38 10.1016/0149-7634(94)00020-2 7770195

[B26] O'Doherty J Kringelbach ML Rolls ET Hornak J Andrews C Abstract reward and punishment representations in the human orbitofrontal cortex Nat Neurosci 2001 4 95 102 10.1038/82959 11135651

[B27] Thut G Schultz W Roelcke U Nienhusmeier M Missimer J Maguire RP Leenders KL Activation of the human brain by monetary reward Neuroreport 1997 8 1225 1228 10.1097/00001756-199703240-00033 9175118

[B28] Rolls ET Rolls BJ Kelly PH Shaw SG Wood RJ Dale R The relative attenuation of self-stimulation, eating and drinking produced by dopamine-receptor blockade Psychopharmacologia 1974 38 219 230 10.1007/BF00421374 4423729

[B29] Greene DJ Colich N Iacoboni M Zaidel E Bookheimer SY Dapretto M Atypical neural networks for social orienting in autism spectrum disorders NeuroImage 2011 56 354 362 10.1016/j.neuroimage.2011.02.031 21334443 PMC3091391

[B30] Lin A Adolphs R Rangel A Social and monetary reward learning engage overlapping neural substrates Soc Cogn Affect Neurosci 2012 7 274 281 10.1093/scan/nsr006 21427193 PMC3304477

[B31] Bartels A Zeki S The neural correlates of maternal and romantic love NeuroImage 2004 21 1155 1166 10.1016/j.neuroimage.2003.11.003 15006682

[B32] Phillips ML Bullmore ET Howard R Woodruff PW Wright IC Williams SC Simmons A Andrew C Brammer M David AS Investigation of facial recognition memory and happy and sad facial expression perception: an fMRI study Psychiatry Res 1998 83 127 138 10.1016/S0925-4927(98)00036-5 9849722

[B33] Spreckelmeyer KN Krach S Kohls G Rademacher L Irmak A Konrad K Kircher T Grunder G Anticipation of monetary and social reward differently activates mesolimbic brain structures in men and women Soc Cogn Affect Neurosci 2009 4 158 165 10.1093/scan/nsn051 19174537 PMC2686229

[B34] Jones RM Somerville LH Li J Ruberry EJ Libby V Glover G Voss HU Ballon DJ Casey BJ Behavioral and neural properties of social reinforcement learning J Neurosci 2011 31 13039 13045 10.1523/JNEUROSCI.2972-11.2011 21917787 PMC3303166

[B35] Schmitz N Rubia K van Amelsvoort T Daly E Smith A Murphy DG Neural correlates of reward in autism Br J Psychiatry 2008 192 19 24 10.1192/bjp.bp.107.036921 18174503

[B36] Haznedar MM Buchsbaum MS Wei TC Hof PR Cartwright C Bienstock CA Hollander E Limbic circuitry in patients with autism spectrum disorders studied with positron emission tomography and magnetic resonance imaging Am J Psychiatry 2000 157 1994 2001 10.1176/appi.ajp.157.12.1994 11097966

[B37] Ke X Hong S Tang T Zou B Li H Hang Y Zhou Z Ruan Z Lu Z Tao G Liu Y Voxel-based morphometry study on brain structure in children with high-functioning autism Neuroreport 2008 19 921 925 10.1097/WNR.0b013e328300edf3 18520994

[B38] Scott-Van Zeeland AA Dapretto M Ghahremani DG Poldrack RA Bookheimer SY Reward processing in autism Autism Res 2010 3 53 67 20437601 10.1002/aur.122 PMC3076289

[B39] Dichter GS Felder JN Green SR Rittenberg AM Sasson NJ Bodfish JW Reward circuitry function in autism spectrum disorders Soc Cogn Affect Neurosci 2010 7 160 172 21148176 10.1093/scan/nsq095 PMC3277365

[B40] Kohls G Peltzer J Herpertz-Dahlmann B Konrad K Differential effects of social and non-social reward on response inhibition in children and adolescents Dev Sci 2009 12 614 625 10.1111/j.1467-7687.2009.00816.x 19635087

[B41] Goldstein RZ Cottone LA Jia Z Maloney T Volkow ND Squires NK The effect of graded monetary reward on cognitive event-related potentials and behavior in young healthy adults Int J Psychophysiol 2006 62 272 279 10.1016/j.ijpsycho.2006.05.006 16876894 PMC2424251

[B42] Kohls G Peltzer J Schulte-Ruther M Kamp-Becker I Remschmidt H Herpertz-Dahlmann B Konrad K Atypical brain responses to reward cues in autism as revealed by event-related potentials J Autism Dev Disord 2011 41 1523 1533 10.1007/s10803-011-1177-1 21290174

[B43] Groen Y Wijers AA Mulder LJ Waggeveld B Minderaa RB Althaus M Error and feedback processing in children with ADHD and children with Autistic Spectrum Disorder: an EEG event-related potential study Clin Neurophysiol 2008 119 2476 2493 10.1016/j.clinph.2008.08.004 18824404

[B44] Larson MJ South M Krauskopf E Clawson A Crowley MJ Feedback and reward processing in high-functioning autism Psychiatry Res 2011 187 198 203 10.1016/j.psychres.2010.11.006 21122921

[B45] Vlamings PH Jonkman LM Hoeksma MR van Engeland H Kemner C Reduced error monitoring in children with autism spectrum disorder: an ERP study Eur J Neurosci 2008 28 399 406 10.1111/j.1460-9568.2008.06336.x 18702711

[B46] South M Larson MJ Krauskopf E Clawson A Error processing in high-functioning Autism Spectrum Disorders Biol Psychol 2010 85 242 251 10.1016/j.biopsycho.2010.07.009 20654684

[B47] Henderson H Schwartz C Mundy P Burnette C Sutton S Zahka N Pradella A Response monitoring, the error-related negativity, and differences in social behavior in autism Brain Cogn 2006 61 96 109 10.1016/j.bandc.2005.12.009 16458401 PMC2652868

[B48] Dawson G Munson J Estes A Osterling J McPartland J Toth K Carver L Abbott R Neurocognitive function and joint attention ability in young children with autism spectrum disorder versus developmental delay Child Dev 2002 73 345 358 10.1111/1467-8624.00411 11949896

[B49] Munson J Faja S Meltzoff A Abbott R Dawson G Neurocognitive predictors of social and communicative developmental trajectories in preschoolers with autism spectrum disorders J Int Neuropsychol Soc 2008 14 956 966 10.1017/S1355617708081393 18954476 PMC2978065

[B50] Faja S Murias M Beauchaine T Dawson G Electrodermal Responding to Reward Feedback During Decision Making among High Functioning Children with Autism Spectrum Disorders under review 10.1002/aur.1307 PMC4104611 23893954

[B51] Meyer-Lindenberg A Domes G Kirsch P Heinrichs M Oxytocin and vasopressin in the human brain: social neuropeptides for translational medicine Nat Rev Neurosci 2011 12 524 538 10.1038/nrn3044 21852800

[B52] Ring RH The central vasopressinergic system: examining the opportunities for psychiatric drug development Curr Pharm Des 2005 11 205 225 10.2174/1381612053382241 15638758

[B53] Insel TR The challenge of translation in social neuroscience: a review of oxytocin, vasopressin, and affiliative behavior Neuron 2010 65 768 779 10.1016/j.neuron.2010.03.005 20346754 PMC2847497

[B54] Skuse DH Gallagher L Dopaminergic-neuropeptide interactions in the social brain Trends Cogn Sci 2009 13 27 35 10.1016/j.tics.2008.09.007 19084465

[B55] Gordon I Martin C Feldman R Leckman JF Oxytocin and social motivation Dev Cogn Neurosci 2011 1 471 493 10.1016/j.dcn.2011.07.007 21984889 PMC3185363

[B56] Strathearn L Fonagy P Amico J Montague PR Adult attachment predicts maternal brain and oxytocin response to infant cues Neuropsychopharmacology 2009 34 2655 2666 10.1038/npp.2009.103 19710635 PMC3041266

[B57] Febo M Numan M Ferris CF Functional magnetic resonance imaging shows oxytocin activates brain regions associated with mother-pup bonding during suckling J Neurosci 2005 25 11637 11644 10.1523/JNEUROSCI.3604-05.2005 16354922 PMC6726012

[B58] Riem MM van Ijzendoorn MH Tops M Boksem MA Rombouts SA Bakermans-Kranenburg MJ No Laughing Matter: Intranasal Oxytocin Administration Changes Functional Brain Connectivity during Exposure to Infant Laughter Neuropsychopharmacology 2012 37 1257 1266 10.1038/npp.2011.313 22189289 PMC3306887

[B59] Tost H Kolachana B Hakimi S Lemaitre H Verchinski BA Mattay VS Weinberger DR Meyer-Lindenberg A A common allele in the oxytocin receptor gene (OXTR) impacts prosocial temperament and human hypothalamic-limbic structure and function Proc Natl Acad Sci USA 2010 107 13936 13941 10.1073/pnas.1003296107 20647384 PMC2922278

[B60] Modahl C Green L Fein D Morris M Waterhouse L Feinstein C Levin H Plasma oxytocin levels in autistic children Biol Psychiatry 1998 43 270 277 10.1016/S0006-3223(97)00439-3 9513736

[B61] Green L Fein D Modahl C Feinstein C Waterhouse L Morris M Oxytocin and autistic disorder: alterations in peptide forms Biol Psychiatry 2001 50 609 613 10.1016/S0006-3223(01)01139-8 11690596

[B62] Ylisaukko-oja T Alarcon M Cantor RM Auranen M Vanhala R Kempas E von Wendt L Jarvela I Geschwind DH Peltonen L Search for autism loci by combined analysis of Autism Genetic Resource Exchange and Finnish families Ann Neurol 2006 59 145 155 10.1002/ana.20722 16288458

[B63] Skuse DH Gallagher L Genetic influences on social cognition Pediatr Res 2011 69 85R 91R 10.1203/PDR.0b013e318212f562 21289535

[B64] Tansey KE Brookes KJ Hill MJ Cochrane LE Gill M Skuse D Correia C Vicente A Kent L Gallagher L Anney RJ Oxytocin receptor (OXTR) does not play a major role in the aetiology of autism: genetic and molecular studies Neurosci Lett 2010 474 163 167 10.1016/j.neulet.2010.03.035 20303388

[B65] Jacob S Brune CW Carter CS Leventhal BL Lord C Cook EH Jr Association of the oxytocin receptor gene (OXTR) in Caucasian children and adolescents with autism Neurosci Lett 2007 417 6 9 10.1016/j.neulet.2007.02.001 17383819 PMC2705963

[B66] Wu S Jia M Ruan Y Liu J Guo Y Shuang M Gong X Zhang Y Yang X Zhang D Positive association of the oxytocin receptor gene (OXTR) with autism in the Chinese Han population Biol Psychiatry 2005 58 74 77 10.1016/j.biopsych.2005.03.013 15992526

[B67] Liu X Kawamura Y Shimada T Otowa T Koishi S Sugiyama T Nishida H Hashimoto O Nakagami R Tochigi M Umekage T Kano Y Miyagawa T Kato N Tokunaga K Sasaki T Association of the oxytocin receptor (OXTR) gene polymorphisms with autism spectrum disorder (ASD) in the Japanese population J Hum Genet 2010 55 137 141 10.1038/jhg.2009.140 20094064

[B68] Campbell DB Datta D Jones ST Batey Lee E Sutcliffe JS Hammock EA Levitt P Association of oxytocin receptor (OXTR) gene variants with multiple phenotype domains of autism spectrum disorder J Neurodev Disord 2011 3 101 112 10.1007/s11689-010-9071-2 21484202 PMC3113442

[B69] Lerer E Levi S Salomon S Darvasi A Yirmiya N Ebstein RP Association between the oxytocin receptor (OXTR) gene and autism: relationship to Vineland Adaptive Behavior Scales and cognition Mol Psychiatry 2008 13 980 988 10.1038/sj.mp.4002087 17893705

[B70] Munesue T Yokoyama S Nakamura K Anitha A Yamada K Hayashi K Asaka T Liu HX Jin D Koizumi K Islam MS Huang JJ Ma WJ Kim UH Kim SJ Park K Kim D Kikuchi M Ono Y Nakatani H Suda S Miyachi T Hirai H Salmina A Pichugina YA Soumarkov AA Takei N Mori N Tsuji M Sugiyama T Two genetic variants of CD38 in subjects with autism spectrum disorder and controls Neurosci Res 2010 67 181 191 10.1016/j.neures.2010.03.004 20435366

[B71] Thanseem I Anitha A Nakamura K Suda S Iwata K Matsuzaki H Ohtsubo M Ueki T Katayama T Iwata Y Suzuki K Minoshima S Mori N Elevated transcription factor specificity protein 1 in autistic brains alters the expression of autism candidate genes Biol Psychiatry 2012 71 410 418 10.1016/j.biopsych.2011.09.020 22030357

[B72] Gregory SG Connelly JJ Towers AJ Johnson J Biscocho D Markunas CA Lintas C Abramson RK Wright HH Ellis P Langford CF Worley G Delong GR Murphy SK Cuccaro ML Persico A Pericak-Vance MA Genomic and epigenetic evidence for oxytocin receptor deficiency in autism BMC Med 2009 7 62 10.1186/1741-7015-7-62 19845972 PMC2774338

[B73] MacDonald E Dadds MR Brennan JL Williams K Levy F Cauchi AJ A review of safety, side-effects and subjective reactions to intranasal oxytocin in human research Psychoneuroendocrinology 2011 36 1114 1126 10.1016/j.psyneuen.2011.02.015 21429671

[B74] Domes G Lischke A Berger C Grossmann A Hauenstein K Heinrichs M Herpertz SC Effects of intranasal oxytocin on emotional face processing in women Psychoneuroendocrinology 2010 35 83 93 10.1016/j.psyneuen.2009.06.016 19632787

[B75] Hurlemann R Patin A Onur OA Cohen MX Baumgartner T Metzler S Dziobek I Gallinat J Wagner M Maier W Kendrick KM Oxytocin enhances amygdala-dependent, socially reinforced learning and emotional empathy in humans J Neurosci 2010 30 4999 5007 10.1523/JNEUROSCI.5538-09.2010 20371820 PMC6632777

[B76] Bartz JA Zaki J Bolger N Hollander E Ludwig NN Kolevzon A Ochsner KN Oxytocin selectively improves empathic accuracy Psychol Sci 2010 21 1426 1428 10.1177/0956797610383439 20855907 PMC6634294

[B77] Rimmele U Hediger K Heinrichs M Klaver P Oxytocin makes a face in memory familiar J Neurosci 2009 29 38 42 10.1523/JNEUROSCI.4260-08.2009 19129382 PMC6664913

[B78] Kosfeld M Heinrichs M Zak PJ Fischbacher U Fehr E Oxytocin increases trust in humans Nature 2005 435 673 676 10.1038/nature03701 15931222

[B79] Buchheim A Heinrichs M George C Pokorny D Koops E Henningsen P O'Connor MF Gundel H Oxytocin enhances the experience of attachment security Psychoneuroendocrinology 2009 34 1417 1422 10.1016/j.psyneuen.2009.04.002 19457618 PMC3138620

[B80] Declerck CH Boone C Kiyonari T Oxytocin and cooperation under conditions of uncertainty: the modulating role of incentives and social information Horm Behav 2010 57 368 374 10.1016/j.yhbeh.2010.01.006 20080100

[B81] Modi ME Young LJ The oxytocin system in drug discovery for autism: Animal models and novel therapeutic strategies Horm Behav 2012 61 340 350 10.1016/j.yhbeh.2011.12.010 22206823 PMC3483080

[B82] Hollander E Bartz J Chaplin W Phillips A Sumner J Soorya L Anagnostou E Wasserman S Oxytocin increases retention of social cognition in autism Biol Psychiatry 2007 61 498 503 10.1016/j.biopsych.2006.05.030 16904652

[B83] Andari E Duhamel JR Zalla T Herbrecht E Leboyer M Sirigu A Promoting social behavior with oxytocin in high-functioning autism spectrum disorders Proc Natl Acad Sci USA 2010 107 4389 4394 10.1073/pnas.0910249107 20160081 PMC2840168

[B84] Guastella AJ Einfeld SL Gray KM Rinehart NJ Tonge BJ Lambert TJ Hickie IB Intranasal oxytocin improves emotion recognition for youth with autism spectrum disorders Biol Psychiatry 2010 67 692 694 10.1016/j.biopsych.2009.09.020 19897177

[B85] Della Libera C Chelazzi L Learning to attend and to ignore is a matter of gains and losses Psychol Sci 2009 20 778 784 10.1111/j.1467-9280.2009.02360.x 19422618

[B86] Raymond JE O'Brien JL Selective visual attention and motivation: the consequences of value learning in an attentional blink task Psychol Sci 2009 20 981 988 10.1111/j.1467-9280.2009.02391.x 19549080

[B87] Krebs RM Boehler CN Woldorff MG The influence of reward associations on conflict processing in the Stroop task Cognition 2010 117 341 347 10.1016/j.cognition.2010.08.018 20864094 PMC2967668

[B88] Hickey C Chelazzi L Theeuwes J Reward guides vision when it's your thing: trait reward-seeking in reward-mediated visual priming PLoS One 2010 5 e14087 10.1371/journal.pone.0014087 21124893 PMC2990710

[B89] Hickey C Chelazzi L Theeuwes J Reward changes salience in human vision via the anterior cingulate J Neurosci 2010 30 11096 11103 10.1523/JNEUROSCI.1026-10.2010 20720117 PMC6633486

[B90] Della Libera C Chelazzi L Visual selective attention and the effects of monetary rewards Psychol Sci 2006 17 222 227 10.1111/j.1467-9280.2006.01689.x 16507062

[B91] Peck CJ Jangraw DC Suzuki M Efem R Gottlieb J Reward modulates attention independently of action value in posterior parietal cortex J Neurosci 2009 29 11182 11191 10.1523/JNEUROSCI.1929-09.2009 19741125 PMC2778240

[B92] Serences JT Value-based modulations in human visual cortex Neuron 2008 60 1169 1181 10.1016/j.neuron.2008.10.051 19109919 PMC3384552

[B93] Navalpakkam V Koch C Rangel A Perona P Optimal reward harvesting in complex perceptual environments Proc Natl Acad Sci USA 2010 107 5232 5237 10.1073/pnas.0911972107 20194768 PMC2841865

[B94] Pessoa L Engelmann JB Embedding reward signals into perception and cognition Front Neurosci 2010 4 17 20859524 10.3389/fnins.2010.00017 PMC2940450

[B95] Anderson BA Laurent PA Yantis S Learned value magnifies salience-based attentional capture PLoS One 2011 6 e27926 10.1371/journal.pone.0027926 22132170 PMC3221688

[B96] Anderson BA Laurent PA Yantis S Value-driven attentional capture Proc Natl Acad Sci USA 2011 108 10367 10371 10.1073/pnas.1104047108 21646524 PMC3121816

[B97] Vismara LA Rogers SJ Behavioral treatments in autism spectrum disorder: what do we know? Annu Rev Clin Psychol 2010 6 447 468 10.1146/annurev.clinpsy.121208.131151 20192785

[B98] Landa RJ Holman KC O'Neill AH Stuart EA Intervention targeting development of socially synchronous engagement in toddlers with autism spectrum disorder: a randomized controlled trial J Child Psychol Psychiatry 2011 52 13 21 10.1111/j.1469-7610.2010.02288.x 21126245 PMC3059234

[B99] Ingersoll B Pilot randomized controlled trial of Reciprocal Imitation Training for teaching elicited and spontaneous imitation to children with autism J Autism Dev Disord 2010 40 1154 1160 10.1007/s10803-010-0966-2 20155309 PMC3686149

[B100] Kasari C Gulsrud AC Wong C Kwon S Locke J Randomized controlled caregiver mediated joint engagement intervention for toddlers with autism J Autism Dev Disord 2010 40 1045 1056 10.1007/s10803-010-0955-5 20145986 PMC2922697

[B101] Dawson G Toth K Abbott R Osterling J Munson J Estes A Liaw J Early social attention impairments in autism: social orienting, joint attention, and attention to distress Dev Psychol 2004 40 271 283 14979766 10.1037/0012-1649.40.2.271

[B102] Mosconi MW Cody-Hazlett H Poe MD Gerig G Gimpel-Smith R Piven J Longitudinal study of amygdala volume and joint attention in 2- to 4-year-old children with autism Arch Gen Psychiatry 2009 66 509 516 10.1001/archgenpsychiatry.2009.19 19414710 PMC3156446

[B103] Hessl D Berry-Kravis E Cordeiro L Yuhas J Ornitz EM Campbell A Chruscinski E Hervey C Long JM Hagerman RJ Prepulse inhibition in fragile X syndrome: feasibility, reliability, and implications for treatment Am J Med Genet B Neuropsychiatr Genet 2009 150B 545 553 10.1002/ajmg.b.30858 18785205 PMC2693303

[B104] Guiraud JA Kushnerenko E Tomalski P Davies K Ribeiro H Johnson MH Differential habituation to repeated sounds in infants at high risk for autism Neuroreport 2011 22 845 849 21934535 10.1097/WNR.0b013e32834c0bec

[B105] Dawson G Webb SJ Carver L Panagiotides H McPartland J Young children with autism show atypical brain responses to fearful versus neutral facial expressions of emotion Dev Sci 2004 7 340 359 10.1111/j.1467-7687.2004.00352.x 15595374

[B106] Webb SJ Dawson G Bernier R Panagiotides H ERP evidence of atypical face processing in young children with autism J Autism Dev Disord 2006 36 881 890 10.1007/s10803-006-0126-x 16897400 PMC2989721

[B107] McPartland J Dawson G Webb SJ Panagiotides H Carver LJ Event-related brain potentials reveal anomalies in temporal processing of faces in autism spectrum disorder J Child Psychol Psychiatry 2004 45 1235 1245 10.1111/j.1469-7610.2004.00318.x 15335344

[B108] Faja S Webb SJ Jones E Merkle K Kamara D Bavaro J Aylward E Dawson G The effects of face expertise training on the behavioral performance and brain activity of adults with high functioning autism spectrum disorders J Autism Dev Disord 2012 42 278 293 10.1007/s10803-011-1243-8 21484517 PMC3707515

[B109] Klin A Jones W Schultz R Volkmar F Cohen D Visual fixation patterns during viewing of naturalistic social situations as predictors of social competence in individuals with autism Arch Gen Psychiatry 2002 59 809 816 10.1001/archpsyc.59.9.809 12215080

[B110] Shultz S Klin A Jones W Inhibition of eye blinking reveals subjective perceptions of stimulus salience Proc Natl Acad Sci USA 2011 108 21270 21275 10.1073/pnas.1109304108 22160686 PMC3248475

